# Association Between Gastroesophageal Reflux Disease and Hypertension: A Systematic Review and Meta‐Analysis

**DOI:** 10.1002/jgh3.70158

**Published:** 2025-04-12

**Authors:** Ganesh Bushi, Abhay M. Gaidhane, Nasir Vadia, Soumya V. Menon, Kattela Chennakesavulu, Rajashree Panigrahi, Muhammed Shabil, Diptismita Jena, Harish Kumar, Anju Rani, Sanjit Sah, Mahendra Pratap Singh, Swastik Subhankar Sahu, Suraj Tiwari, Khang Wen Goh

**Affiliations:** ^1^ Chitkara Centre for Research and Development Chitkara University Baddi India; ^2^ Faculty of Data Science and Information Technology INTI International University Nilai Malaysia; ^3^ Jawaharlal Nehru Medical College, and Global Health Academy, School of Epidemiology and Public Health. Datta Meghe Institute of Higher Education Wardha India; ^4^ Marwadi University Research Center, Department of Pharmaceutical Sciences Faculty of Health Sciences, Marwadi University Rajkot India; ^5^ Department of Chemistry and Biochemistry School of Sciences, JAIN (Deemed to be University) Bangalore India; ^6^ Department of Chemistry Sathyabama Institute of Science and Technology Chennai India; ^7^ Department of Microbiology IMS and SUM Hospital, Siksha'O' Anusandhan (Deemed to be University) Bhubaneswar India; ^8^ University Center for Research and Development Chandigarh University Mohali India; ^9^ School of Pharmaceutical Sciences Lovely Professional University Phagwara India; ^10^ Centre of Research Impact and Outcome Chitkara University Rajpura India; ^11^ Division of Research and Innovation Uttaranchal University Dehradun India; ^12^ New Delhi Institute of Management, Tughlakabad Institutional Area New Delhi India; ^13^ Department of Microbiology Graphic Era (Deemed to be University) Dehradun India; ^14^ SR Sanjeevani Hospital Siraha Nepal; ^15^ Department of Public Health Dentistry Dr. D. Y. Patil Medical College Hospital and Research Centre, Dr. D. Y. Patil Vidyapeeth (Deemed to be University) Pune India; ^16^ Department of Medicine Korea Universtiy Seoul South Korea; ^17^ Center for Global Health Research, Saveetha Medical College and Hospital, Saveetha Institute of Medical and Technical Sciences, Saveetha University Chennai India; ^18^ Noida Institute of Engineering and Technology (Pharmacy Institute) Greater Noida India; ^19^ Department of Pharmacy Practice National Institute of Pharmaceutical Education and Research Guwahati India; ^20^ Faculty of Mathematics and Natural Sciences Universitas Negeri Padang Padang Indonesia

**Keywords:** gastroesophageal reflux disease, hypertension, meta‐analysis, systematic review

## Abstract

**Background:**

Gastroesophageal reflux disease (GERD) and hypertension (HTN) are common conditions with substantial health burdens. While prior studies suggest a link between GERD and HTN, findings remain inconsistent. This systematic review and meta‐analysis aimed to clarify the relationship between GERD and HTN.

**Method:**

A systematic search of PubMed, Web of Science, and Embase was conducted to identify observational studies published through December 2024. Studies evaluating the prevalence and association between GERD and HTN were included. Screening and data extraction were performed using Nested Knowledge software, with quality assessed via the Modified Newcastle–Ottawa Scale. Random‐effects meta‐analyses were used to calculate pooled prevalence and risk estimates, while heterogeneity was quantified using the *I* ^2^ statistic. Publication bias was evaluated using DOI and funnel plots.

**Results:**

Twelve studies, with sample sizes ranging from 71 to 12  960 participants, were included. The pooled prevalence of HTN among GERD patients was 16.80% (95% CI: 6.28%–31.02%) with high heterogeneity (*I* ^2^ = 99%). The pooled risk ratio (RR) for HTN was 1.381 (95% CI: 0.992–1.922) and heterogeneity (*I* ^2^ = 76%) highlighted variability. Sensitivity analyses and publication bias were evident.

**Conclusion:**

GERD is a potential risk factor for HTN, with GERD patients demonstrating an elevated likelihood of developing HTN. Future research is required to investigate the underlying mechanisms and confounding factors that may influence this association.

## 1 Introduction

Gastroesophageal reflux disease (GERD) is a prevalent condition, affecting approximately 20% of the adult population in developed countries [1]. It is characterized by symptoms such as heartburn and acid regurgitation, resulting from the backflow of stomach acid into the esophagus [2]. While GERD is primarily a gastrointestinal disorder, it is increasingly recognized for its association with various comorbidities, including cardiovascular diseases like hypertension (HTN) [3]. However, the relationship between GERD and HTN remains unclear, with studies showing inconsistent findings—some suggest a positive association, while others report no significant link or even a potential protective effect of GERD on blood pressure [4].

HTN is a leading cause of cardiovascular morbidity and mortality worldwide, affecting about 30% of the global population [5, 6]. Given the widespread prevalence of both conditions, understanding whether GERD contributes to the development or exacerbation of HTN is crucial. Potential mechanisms include the systemic inflammatory responses linked to GERD, which may influence vascular function, as well as the effects of GERD medications, such as proton pump inhibitors (PPIs), on blood pressure regulation [7]. Additionally, common risk factors, such as obesity, smoking, and poor diet, may confound the association between GERD and HTN, complicating the interpretation of study results [8].

This systematic review and meta‐analysis aim to clarify the relationship between GERD and HTN by synthesizing evidence from cohort studies and case–control studies. By examining the strength and direction of the association while accounting for potential confounders, we aim to provide a more reliable understanding of how GERD may influence the risk of developing HTN. Given the substantial burden of both conditions, this analysis has the potential to guide clinical management and inform future research directions, ultimately contributing to improved patient outcomes.

## 2 Methods

This meta‐analysis was carried out in accordance with the PRISMA guidelines (Table S1) [9], and the study protocol was registered in the PROSPERO database **(CRD42024618497)**.

### 2.1 Eligibility Criteria

The study focuses on individuals diagnosed with GERD and HTN. Eligible studies must report on the prevalence, incidence, and severity of GERD and HTN, as well as the association between these conditions. The studies should be quantitative in nature, including observational designs such as cohort, case–control, and cross‐sectional studies, and must be published in English. Studies will be excluded if they do not assess the relationship between GERD and HTN, or if they focus on other gastrointestinal or cardiovascular diseases. Other exclusions include studies without relevant control groups, studies without specified outcomes related to GERD and HTN, as well as case series, letters to the editor, commentaries, abstract‐only articles, case reports, review articles, and discussion papers. Studies with unavailable full‐text articles will also be excluded. These criteria aim to ensure a focused analysis of the relationship between GERD and HTN (Table S2).

### 2.2 Search Strategy

A comprehensive literature search was conducted up to December 2024 across PubMed, Web of Science, and Embase electronic databases, utilizing a combination of keywords and Medical Subject Headings (MeSH) terms related to GERD and HTN. The search terms included: “Gastroesophageal reflux disease,” or “Reflux esophagitis,” or “Erosive esophagitis,” in conjunction with “Hypertension,” or “High blood pressure,” or “Elevated blood pressure.” A detailed search strategy for each database is provided in Table S3.

### 2.3 Screening and Data Extraction

The screening and data extraction were conducted using the Nested Knowledge software. Two independent authors evaluated the articles and extracted data. In case of disagreements, a third author was consulted. The process was carried out in two stages: initially, titles and abstracts were reviewed to exclude studies that did not meet the inclusion criteria, followed by a full‐text review to confirm the eligibility of the remaining studies. Disagreements were resolved through discussion, and if needed, a third reviewer made the final decision. After identifying eligible studies, data extraction was performed using a structured tagging method. The following information was recorded: study design, sample size, participant characteristics, and outcomes related to GERD and HTN (e.g., prevalence, effect size [HR/OR/RR], confidence intervals). All extracted data were cross‐verified to ensure accuracy and consistency.

### 2.4 Quality Assessment

The quality of the included studies was assessed using the Modified Newcastle–Ottawa Scale (NOS), which is commonly used to evaluate observational studies in meta‐analyses. This scale assesses three key domains: selection, comparability, and outcome/exposure. Studies are scored from 0 to 6 stars, with scores categorizing studies as high, moderate, or low quality. The scale is adaptable to various study designs, including cross‐sectional studies, though it may involve some level of subjectivity in scoring. It is widely utilized in systematic reviews and meta‐analyses to ensure a comprehensive evaluation of nonrandomized studies, with an emphasis on sample representativeness, control of confounding factors, and the reliability of outcome or exposure measurements. The results of the quality assessment were provided in Table S4 [10].

### 2.5 Evidence Synthesis

The meta‐analysis was conducted using R program version 4.4 [11]. To calculate the prevalence of GERD and HTN, data from multiple studies were pooled. The outcome of the study was the association between HTN and GERD, quantified as pooled effect estimates expressed as adjusted hazard ratios (aHR), with odds ratios (ORs) converted to risk ratios (RRs) before pooling. The *I* ^2^ statistic was employed to evaluate the heterogeneity among the included studies [12, 13].

## 3 Results

### 3.1 Literature Search

**FIGURE 1:**
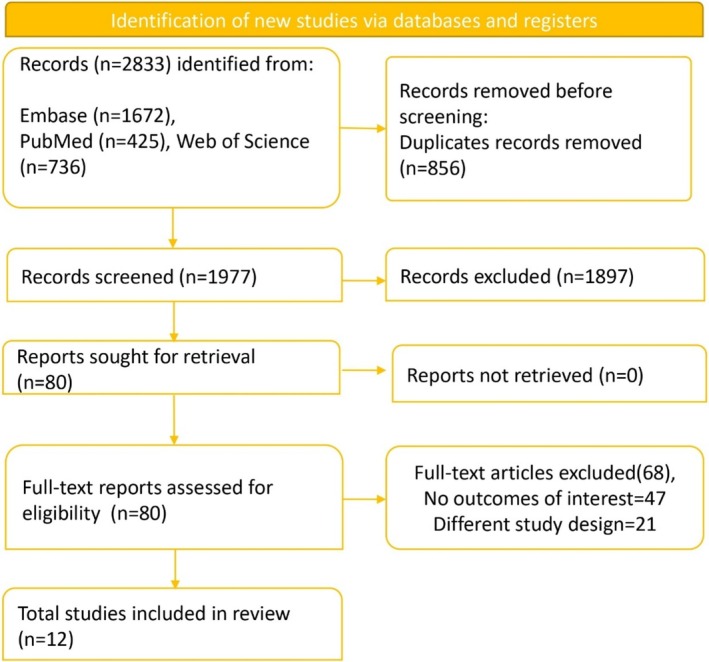
PRISMA flow chart showing the studies selection process.

After a preliminary search, 2833 studies were selected from the database. Excluded 856 duplicate studies and further excluded 1897 irrelevant studies by reading the titles and abstracts of the remaining studies. The full text of the remaining 80 studies was checked, and 68 studies that met the exclusion criteria were excluded. Finally, 12 studies were included in this study (Figure 1).

### 3.2 Summary Characteristics of Included Studies

The 12 studies included in this review were conducted across diverse countries, providing valuable insights into the relationship between GERD and HTN. Among these, two studies were conducted in the United States [14, 15], two in China [16, 17], two in South Korea [18, 19], one in Japan [20], one in Brazil [21], two in India [22, 23], one in Colombia [24], and one in Taiwan [25]. These studies predominantly utilized retrospective cohort designs to explore the association between GERD and HTN, with sample sizes ranging from 71 to 12  960 participants. The study periods varied, covering years from 1988 to 2019. The mean ages of participants ranged from the mid‐30s to early 60s, although one Brazilian study reported an anomalously low mean age of 9.2 years. The proportion of male participants varied significantly across studies, ranging from 22.5% to 71.8%. In terms of outcomes, effect sizes were reported as aHR and OR, indicating varied risks of HTN among GERD patients. The associations ranged from significantly increased risks to less pronounced or statistically insignificant associations (Table 1).

**TABLE 1:** Summary characteristics of included studies.

Study ID	Study design	Study duration	Country	Study population	Mean age (years)	Male %	Sample size	Hypertension events	Effect size
Bunch_2008 [14]	Retrospective cross‐sectional study	1988–1994	United States	Patients with GERD	52	51	2577	592	NA
Chen_2016 [16]	Retrospective cohort study	2000–2011	China	Patient with GERD	48.8	50.8	12 960	3331	HR (95% CI) 2.30 (2.06–2.58)
Eisa_2020 [15]	Retrospective cohort study	NA	United States	Patient with GERD	NA	NA	NA	NA	OR (95% CI) 6.53 (6.21–6.88)
Min_2017 [18]	Retrospective cohort study	2003–2013	South Korea	Patients with reflux esophagitis	53.8	NA	984	78	OR (95% CI) 1.415 (1.263–1.585)
Moki_2007 [20]	Retrospective cohort study	1998–2002	Japan	Patient with GERD	47.1	69.78	5159	862	OR (95% CI) 1.5 (1.06–2.11)
Moraes‐Filho_2009 [21]	Prospective cohort study	September 2006 to December 2007	Brazil	Patient with GERD	9.2	31.4	670	Arterial HTN −344	NA
Nandyal_2017 [22]	Retrospective cohort study	2011–2015	India	Patients with GERD	62.18	50.7	71	12	NA
Quintero_2021 [24]	Retrospective cohort study	January 2016 to June 2017	Colombia	Patients with GERD	45.3	22.5	129	Arterial HTN −2	NA
Sharma_2011 [23]	Retrospective observational study	June 2003 to January 2005	India	Patients with GERD	35.7	71.8	653	47	OR (95% CI) 1.62 (1.20–2.50)
Song_2022 [19]	Retrospective cohort study	2003–2017	South Korea	Patients with GERD	55.6	71	NA	NA	OR 1.173 (1.066–1.290)
Wei_2020 [25]	Retrospective cohort study	2016–2019	Taiwan	GERD patients	NA	NA	NA	102	HR (95% CI) 2.39 (1.49–3.82)
Zhao_2021 [17]	Retrospective cohort study	July 2016 to January 2019	China	Patients with GERD	NA	NA	NA	NA	OR (95% CI) 0.516 (0.219–1.215)

Abbreviations: GERD, gastroesophageal reflux disease; HR, hazard ratio; HTN, hypertension; OR, odd ratio.

### 3.3 Meta‐Analysis

#### 3.3.1 Prevalence of HTN Among GERD Patients

**FIGURE 2:**
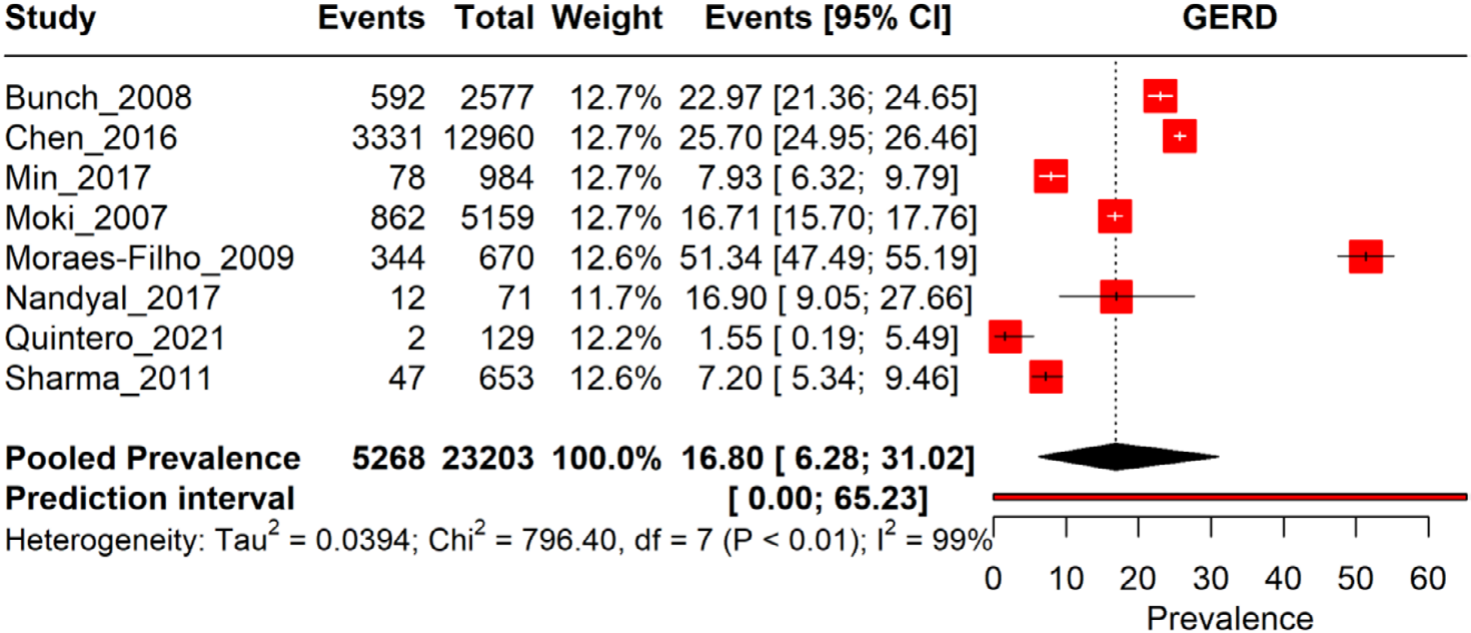
Forest plot illustrating the prevalence of HTN conditions among GERD.

**FIGURE 3:**
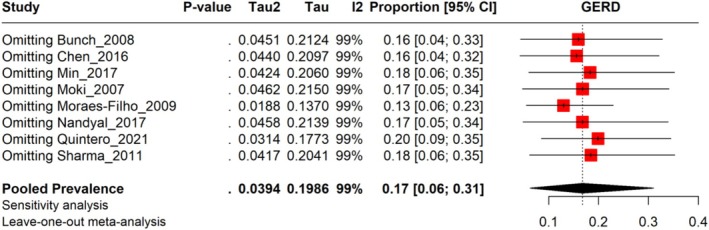
Leave‐one‐out analysis representing results of GERD.

The pooled prevalence of HTN in GERD patients among eight studies is estimated to be 16.80% (95% CI: 6.28%–31.02%), with heterogeneity observed (*I* ^2^ = 99%). The prevalence rates vary widely across the included studies, ranging from 1.55% to 51.34%, depending on study design, sample size, and population characteristics. The prediction interval ranged from 0.00 to 65.23, indicating a wide range of outcomes that depend on disease severity, treatment approaches, and healthcare settings (Figure 2). Leave‐one‐out Sensitivity analysis revealed no studies influenced the pooled results (Figure 3).

#### 3.3.2 Association of HTN Among GERD Patients

**FIGURE 4:**
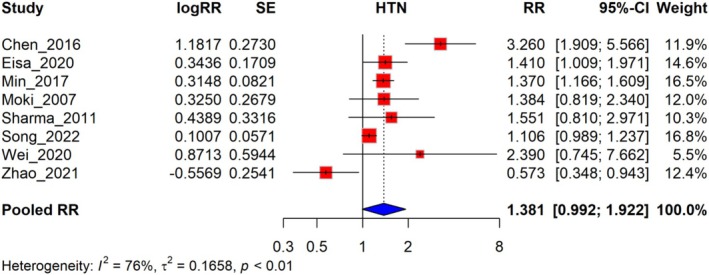
Association of HTN conditions among GERD.

**FIGURE 5:**
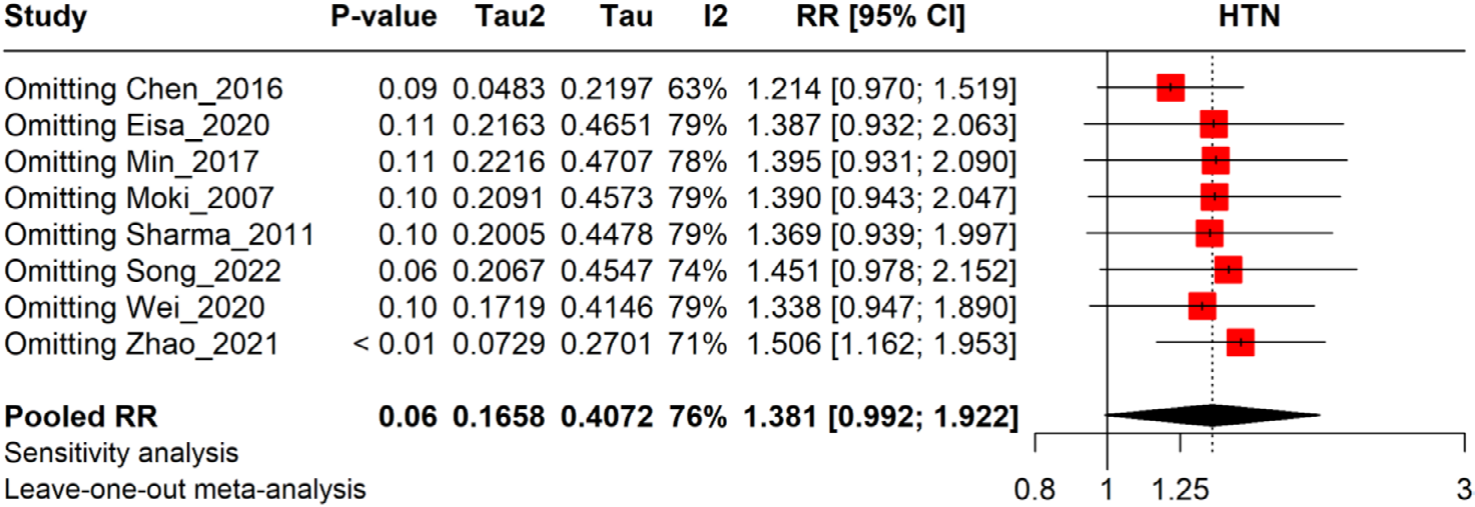
Leave‐one‐out analysis representing risk ratio results of HTN conditions among GERD.

The meta‐analysis indicates that the pooled RR for HTN across the eight studies is 1.381, with a 95% confidence interval (0.992–1.922). Heterogeneity is present among the studies (*I* ^2^ = 76%), indicating substantial variation in the results, which calls for further investigation to identify the sources of this variability. The RR rates ranged from 0.573 to 3.260 across different studies, reflecting a diverse set of clinical settings and patient profiles (Figure 4). A leave‐one‐out sensitivity analysis showed that omitting Chen (2016) from the analysis reduced the pooled RR to 1.214, while omitting Zhao (2021) changed the pooled RR to 1.506. The RR rates ranged from 1.214 to 1.506 across different studies, reflecting a diverse set of clinical settings and patient profiles (Figure 5).

#### 3.3.3 Publication Bias

The assessment of publication bias using DOI plots shows notable asymmetry, with LFK index values of −3.18 (Figure S5). Values outside the range of ± 2 typically indicate significant asymmetry, which suggests considerable publication bias among the studies. This bias likely leads to the underreporting of smaller or nonsignificant studies, potentially distorting the pooled effect estimates and undermining the validity of the meta‐analytic conclusions. Addressing this bias is crucial to enhancing the reliability and applicability of the findings [26, 27]. Publication bias was also evaluated using a funnel plot, with RR rates ranging from 1.381 to 1.922 across various studies, reflecting a wide range of clinical settings and patient demographics (Figure S6). The funnel plot visually examines publication bias and small study effects, with the *x*‐axis displaying effect size and the *y*‐axis showing precision (inverse of standard error). Asymmetry in the plot suggests bias, where smaller studies may be underreported or report more extreme results. Though useful, funnel plots require statistical tests like Egger's test and Trim and Fill to ensure a more accurate interpretation [28].

## 4 Discussion

Our findings suggest a significant, though variable, association between GERD and HTN, highlighting the complexity of this relationship across different populations. The pooled prevalence of HTN among GERD patients was estimated at 16.80% (95% CI: 6.28%–31.02%); heterogeneity (*I* ^2^ = 99%) was observed. This variability reflects differences in study populations, diagnostic criteria, and methodological approaches. The pooled RR for HTN among GERD patients was 1.381 (95% CI: 0.992–1.922), suggesting a potential association, though the wide confidence interval indicates uncertainty. The range of RR values across studies (0.573–3.260) further highlights the influence of study design and population differences on the observed association.

This study supports the relationship between GERD and HTN, highlighting shared inflammatory and metabolic pathways. Our meta‐analysis, which found a pooled RR of 1.381 for HTN among GERD patients, aligns with prior studies suggesting an increased risk of HTN in GERD populations. We identified a causal link through Mendelian randomization, implicating inflammation‐mediated pathways, and highlighted shared inflammatory mechanisms involving cytokines like IL‐6 and TNF‐α that contribute to vascular endothelial dysfunction [7, 8]. However, findings are inconsistent; a Taiwanese cohort study reported no significant association, possibly reflecting variability in population characteristics and diagnostic criteria. We noted that shared risk factors, such as obesity and diet, may confound the relationship, emphasizing the need for robust adjustment in analyses [29]. Our results underscore systemic inflammation as a key mechanism, consistent with findings of HTN exacerbating GERD‐related esophageal damage through immune activation [30]. Additionally, obesity is a critical contributor to both conditions, as highlighted by prior meta‐analyses linking their rising prevalence to increased obesity rates. A unique contribution of this meta‐analysis is quantifying heterogeneity in risk estimates (*I* ^2^ = 76%), emphasizing variability in the GERD‐HTN association due to differences in diagnostic criteria and methodologies. While the evidence supports a link between GERD and HTN, inconsistencies suggest further research is needed using standardized approaches to address study design and confounding variables. This study reinforces the roles of inflammation and shared risk factors while identifying critical gaps in understanding the cardiovascular implications of GERD.

The relationship between GERD and HTN involves a complex interplay of biological mechanisms, medication effects, and lifestyle factors. Key contributors include autonomic nervous system dysfunction, chronic inflammation, and oxidative stress [31]. GERD‐induced vagal nerve activity may influence blood pressure control, while inflammation, driven by cytokines such as IL‐6 and TNF‐α, promotes endothelial dysfunction, a precursor to HTN [32]. Oxidative stress from persistent reflux‐related tissue damage further impairs vascular function. Additionally, medications like PPIs, while effective for managing GERD symptoms, may have cardiovascular implications, such as reduced nitric oxide bioavailability and endothelial health, necessitating careful use in patients with heightened cardiovascular risk [33]. Shared lifestyle factors, including obesity and dietary patterns, further link these conditions. Obesity elevates intra‐abdominal pressure, exacerbating GERD and driving inflammation, while high sodium intake and GERD‐trigger foods can worsen both conditions [29]. These interconnected mechanisms highlight the need for integrated research and clinical care.

Clinicians must prioritize early identification and comprehensive management of these overlapping conditions. GERD patients, particularly those with obesity or metabolic syndrome, benefit from routine blood pressure monitoring, while hypertensive individuals should be evaluated for GERD symptoms to enhance treatment effectiveness [34]. Lifestyle changes, such as weight loss, healthy dietary adjustments, and stress management, address underlying risk factors for both conditions [35]. Weight loss, for instance, relieves GERD symptoms by reducing abdominal pressure and also lowers blood pressure. Medication use requires cautious consideration to prevent aggravating either condition, given the potential risks of PPIs and some antihypertensive drugs. Public health efforts should focus on promoting healthy behaviors, early interventions, and equitable access to care. Campaigns targeting obesity reduction, improved nutrition, and active lifestyles could significantly reduce the burden of these diseases [36]. Enhanced healthcare access and targeted interventions in high‐risk populations will further alleviate the impact of GERD and HTN, benefiting both individuals and healthcare systems globally.

The limitations, including variability in study quality and the observational nature of most studies, should be noted. While the Modified NOS scale was used to assess quality, its inherent subjectivity may not fully capture all biases. The significant heterogeneity observed suggests that future studies should standardize diagnostic criteria and focus on more homogeneous populations to better understand the relationship between GERD and HTN. Given the observational design of most studies, causality cannot be established. Future research should prioritize randomized controlled trials (RCTs) to explore and investigate underlying mechanisms, such as biomarkers or genetic factors, that may predispose individuals to both conditions.

Additionally, evaluating the effectiveness of interventions targeting conditions concurrently could offer new insights into management strategies. While the evidence supports a link between these conditions, the significant heterogeneity and publication bias highlight the need for more rigorous, well‐designed studies to clarify the nature of this relationship. Clinicians should consider monitoring and managing both conditions concurrently, while future research should aim to identify underlying mechanisms and evaluate integrated treatment approaches.

## 5 Conclusion

A potential association between HTN and GERD patients was found. Shared mechanisms, including systemic inflammation, oxidative stress, and lifestyle factors, appear to play a key role in their coexistence. Despite significant heterogeneity and publication bias, these findings highlight the need for integrated management strategies focusing on lifestyle interventions, early screening, and careful medication use to address overlapping risk factors.

## Consent

The authors have nothing to report.

## Conflicts of Interest

The authors declare no conflicts of interest.

## Supporting information


**Table S1.** PRISMA checklist.
**Table S2**. Inclusion and exclusion criteria.
**Table S3**. The adjusted search terms as per searched electronic databases.
**Table S4**. Modified NOS scale for the quality assessment of included studies.
**Figure S5**. Publication bias assessment using DOI plots of HTN associated among GERD.
**Figure S6**. Publication bias assessment using funnel plots of HTN associated among GERD.

## Data Availability

All data generated or analyzed during this study are included in this published article (and its Supporting Information files).
